# An Unclassified Disease Following Parturition in Cows1Read before the twenty-first regular meeting of the Missouri Valley Veterinary Medical Association.

**Published:** 1900-01

**Authors:** O. Verschelden

**Affiliations:** St, Mary’s, Kan.


### AN UNCLASSIFIED DISEASE FOLLOWING PARTURITION IN COWS.[1]

By O. Verschelden, V.S.,
ST, MARY'S, KAN.

Time and again has my attention been called to a certain disease of cattle, especially during the fall and winter of 1897 and spring of 1898, when it prevailed as an enzoötic in my district. I have failed to see it mentioned in any works on cattle pathology at my disposal, and feel, therefore, justified in calling it "an unclassified disease," notwithstanding, perhaps, that many of you have met with it as well as myself. It is with that hope that I present this paper before you this evening. I will refrain from dealing with its probable etiology, leaving such to some of you who are, perhaps, better situated than I to study obscure diseases and go into orignal research. I will simply describe the symptoms as they present themselves to me, also my mode of treatment. I hope, however, to hear the experience of others and to have, by a

[1] Read before the twenty-first regular meeting of the Missouri Valley Veterinary Medical Association.

thorough discussion, more light thrown on this, to me at least, obscure disease.

It attacks cows of all ages and conditions in from three days to as many weeks after parturition. The first symptom usually noticed is a peculiar gait of the hind parts—the animal walks with the hind legs sideways, not unlike the trot of a dog. The general health at this stage is not much altered ; perhaps a capricious appetite, with a slight fever, are all the symptoms ; sometimes, especially if put under proper treatment at once, no graver symptoms develop, but such is not always the case; too frequently in a day or two more the symptoms are aggravated to an alarming extent. In so short a time the flesh seems to have melted from the body which was sleek and fat; the coat is rough and staring; the appetite is entirely gone, and with it rumination ceases; pulse is about 70 or 80, and temperature elevated two to three degrees; feces are hard and covered with mucus. What is peculiar in most cases is the surprising amount of milk secreted, considering the condition of the animal. In all cases which came under my observation the animal was able to get up without or with very little assistance; it would, however, have been an easy matter to push her over after she was up, so weak was she. The predominating symptoms are the incoördination of the hind parts and the rapid emaciation.

My mode of treatment consists in first administering a full dose of physic: Sulph. magnesia, 1 pound; gamboge, ℥j. No matter how weak the patient is this is followed by powders two or three times a day, consisting of sodium bicarb., gentian radix pulv., āā and strychnine sulph., grs. iij, combined or alternated in very bad cases with arsen. ac., grs. v. All of my cases so treated recovered in from ten days to two weeks, and once convalescence was established they gained quickly their former condition.

Now, what factor brings about these phenomena in the parturient cow? Has anyone seen it in the non-parturient animal, or in other females beside cows? These are questions which interest me very much, and I would like to hear you, gentlemen, on the subject.

*Discussion.*

Dr. Moore: Dr. Verschelden's paper is certainly a very interesting one, and he has described a series of cases which are certainly not commonly met with, though possibly I may have seen some such. The symptoms are somewhat similar to those found in cases which I have met, and which I attributed to septic intoxication; but in the cases described the lesions were developed too long after parturition to be ascribed to septic infection.

As you are aware, there often remains after parturition small portions of placenta in which putrefactive changes occur, producing a series of symptoms similar to those described, such as elevation of temperature, staggering gait, general weakness, and rapid emaciation. The treatment employed in cases described in the paper would be quite likely to be beneficial in cases of septic intoxication.

Dr. Bennett: I do not remember to have seen any cases like those described in the paper, and I should be pleased to hear what Dr. Verschelden thinks was the cause in his cases.

Dr. Verschelden: I have not been able to make a careful and satisfactory study of it. I have considered it a species of nervous disease, possibly resulting from stomach troubles. It does not seem possible that it was due to septic infection, as in many cases the deliveries were very easy and the membranes came away promptly. Some cases occurred as long as three weeks after very easy delivery, and one thing peculiar about it, there was a very slight diminution of the milk-supply; there were no vaginal discharges and no straining. It is peculiar that these cases were all seen dnring the fall of 1897 and spring of 1898. Fully 50 per cent. of the cows in St. Mary's, where I practice, suffered from the malady. I did not see any cases in the country. My treatment, as you will note, was nerve stimulant and tonic.

Dr. Wright: Dr. Verschelden asks if any of us has seen similar cases. I will say that I saw something similar in a bitch I owned. I noted her one morning having a staggering gait and disinclination to move about. This condition was ascribed by me to fright, as the dog-catchers had endeavored to secure her. She rapidly emaciated thereafter, being reduced to skin and bones. She was finally given a good purgative, which operated promptly, and after which she made rapid recovery.

Dr. Milnes: I understand from the paper that the essayist thought there was some relation between the case of crotalism of which he read and these cases described, and I would like to know if that is his idea.

Dr. Verschelden: Yes.

Dr. Milnes: As I understand crotalism, it is a disease of the horse only, produced by the poisonous weed crotalia sagittalis in the food.

Dr. Verschelden: I was led to believe from the symptoms that there was some poisonous element in the food of these cattle.

Dr. Moore: I can readily see how the train of symptoms given in these cases would indicate that there was a toxic element in the food, but it seems from the paper that only parturient cows were affected, whereas other animals are likely also to be influenced by poisonous food.

Dr. Milnes: Perhaps it is possible that females in the parturient state are more susceptible than males.

Dr. Moore: That may be the case, but we must not forget that in these animals symptoms developed two or three weeks after parturition.

Dr. Milnes: The symptoms are somewhat similar to those in horses suffering from crotalism, in which there are staggering gait, enfeebled eyesight, emaciation, and loss of appetite, which condition extends over a period from three months to eighteen months. I have known cases to die at the end of three months. Crotalaria sagittalis grows in the Missouri River bottoms, in western Iowa and is acquired by the animals both from feeding in the pastures and from hay.

Dr. Verschelden: All of these cases that I saw were in the town of St. Mary's, being dairy cows. No cases were seen in the country about, and the home cows were fed on slops and refuse of the city mills.

Dr. Ernst: I will state that in Leavenworth some of the people feed mill stuff, consisting of the imperfect kernels of corn, and the chaff, the cob, etc., to the horses and cattle, and it produced a condition called stomach staggers in the horses. I never saw anything of the kind in cattle. It was so well understood that this stomach staggers was produced by this mill stuff that the people no longer feed it to horses, but still use it for feed for the cattle.

